# Supplementary material for: Intrinsic motivation in virtual assistant interaction for fostering spontaneous interactions
Source: PLoS One. 2021 Apr 23;16(4):e0250326. doi: 10.1371/journal.pone.0250326 (PMC8064575; doi:10.1371/journal.pone.0250326)
Supplement: S1 Fig — (PDF) [file pone.0250326.s001.pdf]

S8 Fig. Procedures of the verification experiment of effects of uncertainty.

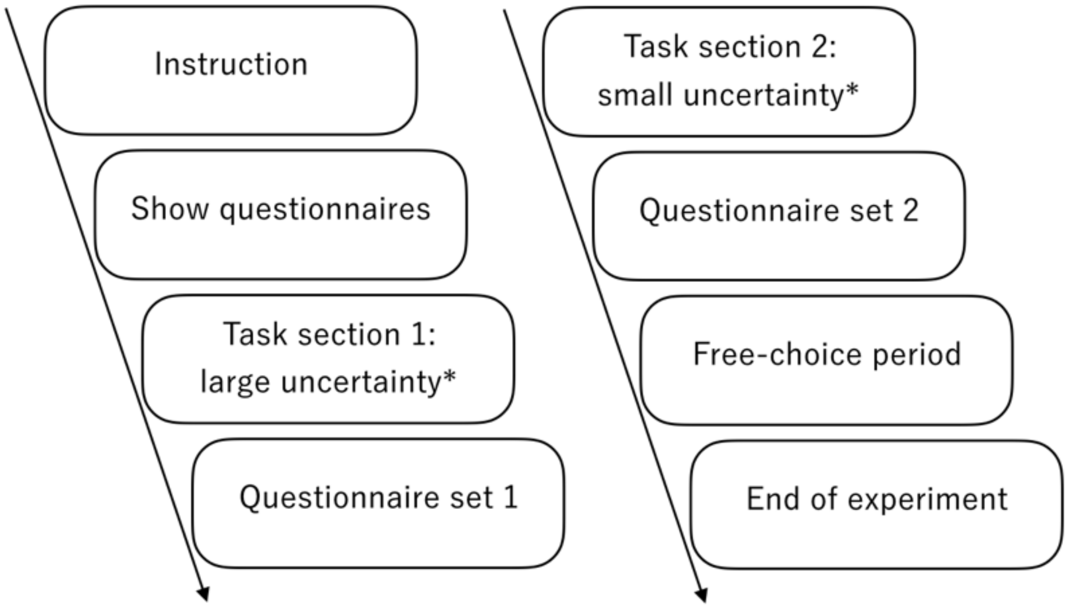

* In order to counterbalance effects of order, half of the participants started with small uncertainty condition and the other half started with large uncertainty condition
