# Supplementary material for: Intrinsic motivation in virtual assistant interaction for fostering spontaneous interactions
Source: PLoS One. 2021 Apr 23;16(4):e0250326. doi: 10.1371/journal.pone.0250326 (PMC8064575; doi:10.1371/journal.pone.0250326)
Supplement: S1 Table — A. Task list for low-expectation conditions. B. Task list for high-expectation conditions. (PDF) [file pone.0250326.s002.pdf]

S1A Table. Task list for low-expectation conditions.

Task #	Task	Example of response by the virtual assistant
1	Roll a 20-sided dice	I rolled a 20-sided dice and got 16
2	Roll two dice	Rolling…5 and 1
3	Roll two 20-sided dice	3 and 11
4	Get a random number between 1 and 6	Your random number between 1 and 6 is 6
5	Get two random numbers between 1 and 6	4 and 4
6	Flip a coin	Tails
7	Roll a dice	I rolled a dice and got 1
8	Flip a coin and then roll a dice	Heads and 2
9	Ask definition of even number	Even number is usually defined as a positive integer that can be divided by two, and two itself. Or, an integer that is divisible by two.
10	Ask whether 5 is an even number	No
11	Get a random even number	372,406 is a random even number
12	Roll a dice and ask if that is an even number	I rolled a dice and got 2. It is an even number

S1B Table. Task list for high-expectation conditions.

Task #	Task	Example of response by the virtual assistant
1	Flip two coins	Tails and tails
2	Roll a dice and flip that many coins	I rolled a dice and got 3. Flipping…Heads, heads, tails.
3	Ask for the number of heads	You’ve got two heads.
4	Turn on the light	<light turned on>
5	Set the light blue	<light turned blue>
6	Set the light to a warm color	<light turned to yellowish white>
7	Set the light's brightness to 50%	<brightness decreases from 100% to 50%>
8	Dim by 30%	<brightness decreases from 50% to 20%>
9	Set the light’s brightness back up	<brightness increases from 20% to 50%>
10	Convert 70,000 Japanese Yen to US dollar	70,000 Japanese Yen is 648 US Dollars and 81 Cents
11	Calculate 1000 US dollars minus 70,000 Japanese Yen	1,000 US Dollars minus 70,000 Japanese Yen is 351 US Dollars and 19 Cents
12	Remember my monthly income: $1000	You want me to remember your income is 1000 US Dollars, right? (upon confirmation, says 'Ok, got it.’)
13	Remember my rent: 70,000 Japanese Yen	Ok, got it.
14	What is my income minus my rent?	The answer is 351 US Dollars and 19 Cents
