# Supplementary material for: Intrinsic motivation in virtual assistant interaction for fostering spontaneous interactions
Source: PLoS One. 2021 Apr 23;16(4):e0250326. doi: 10.1371/journal.pone.0250326 (PMC8064575; doi:10.1371/journal.pone.0250326)
Supplement: S2 Table — A. Response manipulation for low-expectation conditions. B. Response manipulation for high-expectation conditions. (PDF) [file pone.0250326.s003.pdf]

S2A Table. Response manipulation for low-expectation conditions. A circle indicates that the virtual assistant is made to cope with the task, whereas a cross means the virtual assistant is made to give a response such as “Sorry, I don’t know that one,” or “Hmm…I’m not sure about that.” These “can’t do” utterances are borrowed from the actual Amazon Alexa repertoire.

Task #	Small uncertainty	Large uncertainty
1	○	○
2	○	○
3	○	○
4	○	○
5	○	×
6	○	○
7	○	○
8	×	×
9	○	○
10	○	○
11	○	○
12	×	○

S2B Table. Response manipulation for high-expectation conditions.

Task #	Small uncertainty	Large uncertainty
1	○	○
2	○	○
3	○	×
4	○	○
5	○	○
6	○	○
7	○	○
8	○	○
9	×	○
10	○	○
11	○	×
12	○	○
13	○	○
14	×	○
