# Supplementary material for: Intrinsic motivation in virtual assistant interaction for fostering spontaneous interactions
Source: PLoS One. 2021 Apr 23;16(4):e0250326. doi: 10.1371/journal.pone.0250326 (PMC8064575; doi:10.1371/journal.pone.0250326)
Supplement: S3 Table — A. Questionnaire items. B. Questionnaire items by sub-scales. (PDF) [file pone.0250326.s004.pdf]

S3A Table. Questionnaire items.

#	Statement to be rated from 1 (totally disagree) to 7 (totally agree)
1	I enjoyed doing this activity very much.
2	The virtual assistant was stupid.
3	It is likely that the virtual assistant and I could become friends if we interacted a lot.
4	The virtual assistant sounded like a robot.
5	I thought this was a boring activity.
6	I think the virtual assistant would be a useful smart home assistant.
7	The virtual assistant was friendly and easy to talk to.
8	Sometimes the virtual assistant didn’t understand what I said.
9	The virtual assistant has a natural speaking voice.
10	This activity was fun to do.
11	I don’t think that the virtual assistant and I would ever be friends.
12	This activity did not hold my attention at all.
13	The virtual assistant failed to carry out some easy tasks.
14	I don’t feel like I could really trust this virtual assistant.
15	It felt like talking to a real person.
16	I’d like to interact with the virtual assistant more often.
17	I would describe this activity as very interesting.
18	The virtual assistant was not as smart as I had expected.
19	I couldn’t understand what the virtual assistant was thinking.
20	I’d really prefer not to interact with the virtual assistant in the future.
21	While I was doing this activity, I was thinking about how much I enjoyed it.
22	I like the way the virtual assistant talks.
23	I felt like I could really trust the virtual assistant.
24	The virtual assistant’s behaviors were understandable.
25	This activity was quite enjoyable.

S3B Table. Questionnaire items by sub-scales.

Sub-scale	Corresponding questionnaire item #
Intrinsic motivation	1, 5, 10, 12, 17, 21, 25
Smartness	2, 6, 13, 18
Comprehensibility	7, 8, 19, 24
Trust/relatedness	3, 11, 14, 16, 20, 23
Human-likeness	4, 9, 15, 22
