# Supplementary material for: Intrinsic motivation in virtual assistant interaction for fostering spontaneous interactions
Source: PLoS One. 2021 Apr 23;16(4):e0250326. doi: 10.1371/journal.pone.0250326 (PMC8064575; doi:10.1371/journal.pone.0250326)
Supplement: S4 Table — A. Tasks for small-uncertainty condition (Verification experiment of effects of uncertainty). B. Tasks for large-uncertainty condition (Verification experiment of effects of uncertainty). (PDF) [file pone.0250326.s005.pdf]

S6A Table. Tasks for small-uncertainty condition (verification experiment of effects of uncertainty). The virtual assistant was made to appear unable to cope with tasks 3, 7, 9 and 12, but offered informative responses.

Task #	Task	Example of response by the virtual assistant
1	Roll a dice	I rolled a dice and got 6
2	Flip a coin	Heads
3	Roll a dice, and flip that many coins	Sorry, I can’t do it all at once
4	Set the light blue	<light turned on blue>
5	Set the light's brightness to 50%	<brightness adjusted from 100% to 50%>
6	Dim by 30%	<brightness decreased from 50% to 30%>
7	Set the light’s brightness to original level	Sorry, I can't store previous states of the device: light
8	Convert 70,000 Japanese Yen to US dollar	70,000 Japanese Yen is 684 US Dollars and 81 Cents
9	Calculate 1000 US dollars minus 70,000 Japanese Yen	Sorry, I can’t calculate between different currencies
10	Ask the virtual assistant to remember your monthly income: 400,000 JPY	You want me to remember your income is 400,000 Japanese Yen, right? (upon confirmation, says 'Ok, got it.’)
11	Ask the virtual assistant to remember your rent: 110,000 Japanese Yen	Ok, got it
12	Calculate: Income - Rent = ?	Sorry, calculation with variables is not supported yet

S6B Table. Tasks for large-uncertainty condition (verification experiment of effects of uncertainty). The assistant was made to appear unable to cope with tasks 3, 7, 9 and 12, and these responses were not informative.

Task #	Task	Example of response by the virtual assistant
1	Ask for a random even number.	372,406 is a random even number
2	Ask whether 5 is an even number	No
3	Ask for a random number and whether it is an even number	Hmm…I'm not sure
4	Set the light's brightness to 70%	<light's brightness adjusted from 100% to 70%>
5	Set the light blue	<light turned blue>
6	Set the light to a warm color	<light turned to yellowish white>
7	Set the light to the previous color	Hmm…I'm not sure
8	Convert 6 hours and 25 minutes to minutes	6 hours and 25 minutes is 385 minutes
9	Subtract 155 minutes from 4 hours	Hmm…I'm not sure
10	Add paper towel to shopping list	Did you say paper towel? (upon confirmation, says 'Ok, got it.')
11	Ask for recommended brand for paper towel	I found this: Presto! Paper Towels
12	Ask for recommended brand for shopping list	Hmm…I'm not sure
