# Supplementary material for: Intrinsic motivation in virtual assistant interaction for fostering spontaneous interactions
Source: PLoS One. 2021 Apr 23;16(4):e0250326. doi: 10.1371/journal.pone.0250326 (PMC8064575; doi:10.1371/journal.pone.0250326)
Supplement: S5 Table — A. Questionnaire items for the verification experiment of effects of uncertainty. B. Questionnaire items by sub-scales for the verification experiment of effects of uncertainty. (PDF) [file pone.0250326.s006.pdf]

S7A Table. Questionnaire items for the verification experiment of effects of uncertainty.

#	Statement to be rated from 1 (totally disagree) to 7 (totally agree)
1	I would describe this activity as very interesting.
2	I believe this virtual assistant could be a useful smart home assistant.
3	I don't understand what the virtual assistant was thinking.
4	This activity did not hold my attention at all.
5	The virtual assistant failed to perform some easy tasks.
6	This was a boring activity.
7	The thinking process of this virtual assistant was understandable.
8	The virtual assistant was not as smart as I'd expected.
9	I doubt that the virtual assistant understood what I was talking about.
10	I think the virtual assistant is smart.
11	The responses were understandable.
12	This activity was quite enjoyable.

.

S7B Table. Questionnaire items by sub-scales for the verification experiment of effects of uncertainty.

Sub-scale	Corresponding questionnaire item #
Intrinsic motivation	1, 4, 6, 12
Smartness	2, 5, 8, 10
Comprehensibility	3, 7, 9, 11
