# Supplementary material for: Intrinsic motivation in virtual assistant interaction for fostering spontaneous interactions
Source: PLoS One. 2021 Apr 23;16(4):e0250326. doi: 10.1371/journal.pone.0250326 (PMC8064575; doi:10.1371/journal.pone.0250326)
Supplement: S6 Table — (PDF) [file pone.0250326.s007.pdf]

S9 Table. Protocol of dialog for free-choice period.

Participant #	Target	Utterances	Motivation type
1	Alexa	What does "<participant's name>" mean?	Intrinsic
		Can you translate the word "dance"?	Intrinsic
		Do you speak Japanese?	Non-intrinsic
		Can you translate the sentence for me?	Non-intrinsic
		(in Skill) My name is <participant's name>.	Intrinsic
		Can you translate for me?	Non-intrinsic
		How do you say hi in Japanese?	Intrinsic
		How do you say colors in Japanese?	Intrinsic
		How do you say table in Japanese?	Intrinsic
2	Echo	Do you like Siri?	Intrinsic
	Alexa	Do you like Siri?	Intrinsic
3	Alexa	Play rock paper scissors.	Intrinsic
		Play rock paper scissors.	Intrinsic
		Play rock paper scissors.	Intrinsic
		Play rock paper scissors.	Intrinsic
5	Alexa	Tell me a joke.	Intrinsic
		What's your favorite music?	Intrinsic
		Play me your favorite song.	Intrinsic
		Sing me a song.	Intrinsic
		Sing for me.	Intrinsic
		How far is it from Vienna to Tokyo?	Intrinsic
		How old are you?	Intrinsic
		Where are you born?	Intrinsic
		I'm your father.	Intrinsic
		Tell me a bad joke.	Intrinsic
		Speak German	Intrinsic
		What languages do you speak?	Non-intrinsic
		Set the light blue.	Non-intrinsic

		Set the light to bright white.	Non-intrinsic
6	Alexa	When is your birthday?	Intrinsic
		What's how's the weather tomorrow?	Intrinsic
		Can I see starry sky tonight?	Intrinsic
		Play rock paper scissors.	Intrinsic
		Count from 1 to 10.	Intrinsic
		What's your gender?	Intrinsic
		Play a song.	Intrinsic
		Play Beethoven.	Intrinsic
		Sing a song.	Intrinsic
7	Alexa	Do you marry me?	Intrinsic
		Please sing a song.	Intrinsic
		Please play hard rock music.	Intrinsic
		Please play dance music.	Intrinsic
9	Echo	Play music.	Intrinsic
	Alexa	Play music.	Intrinsic
10	Echo	Tell me a joke.	Intrinsic
		What is the weather in Austria?	Non-intrinsic
		What is the weather in Vienna?	Non-intrinsic
	Alexa	Tell me a joke.	Intrinsic
		What is today's weather?	Intrinsic
		What is the weather in Munich?	Non-intrinsic
		What is the weather in Vienna?	Non-intrinsic
		Where is Vienna?	Intrinsic
		Where is Austria?	Intrinsic
		Turn the light to red.	Intrinsic
		Make the light green.	Intrinsic
		Set the light to violet.	Intrinsic
		What is on my shopping list?	Non-intrinsic
		What is my monthly income?	Non-intrinsic
