# Supplementary material for: Intrinsic motivation in virtual assistant interaction for fostering spontaneous interactions
Source: PLoS One. 2021 Apr 23;16(4):e0250326. doi: 10.1371/journal.pone.0250326 (PMC8064575; doi:10.1371/journal.pone.0250326)
Supplement: S1 Appendix — (PDF) [file pone.0250326.s008.pdf]

S4 Appendix. Comprehensive experimental procedures. In this document, we present detailed experimental procedures to complete Section 3.4 Implementation.

The participant was first greeted by the experimenter, then and given introductions about the smart speakers. They were instructed that the two smart speakers have different wake words, 'Echo' and 'Alexa', and that they are two different voice assistants although their voices are identical. At this stage, the smart speakers were muted, which prevented them from being activated during instruction, since 'Echo' and 'Alexa' would be orally mentioned. Next, the participant was shown the seat from where they will be doing the task section. After that, the experimenter covered the smart speakers with a dense dark curtain and activated their microphones at the same time. Participants were told that the smart speakers were covered by dense dark curtains to preclude favoritism based on appearance, although the actual purpose was to hide the LED rings, which would glow in red when the microphones were deactivated during task sections.

From there, the experimenter proceeded to demonstrate three functions: to check weather, to play rock-paper-scissors and to answer trivia question. Each question was first asked to 'Echo' and then asked again for 'Alexa'. The trivia question was: "What is the brightest star in the sky?", to which the virtual assistant answers "After the Sun, the brightest star to the naked eye is Sirius, also known as the Dog Star due to its position in Canis Major." The experimenter also demonstrated how to interrupt the smart speaker's speech (by saying 'Echo, stop' or 'Alexa, stop.')

After the demonstration, the experimenter would go up to the dark curtain and mute the microphones in order to proceed to introduce the task sections. The participant was shown with the task lists and was instructed that they can use any utterance as long as it conveys the task. In other words, there was no

need to read out the tasks from the lists. Here, the participant was instructed that one must carry out the tasks in the given order and that one should not return to previous tasks. However, the participant was allowed to retry the current task as many times as needed (presumably after the question results in an unexpected response.) Otherwise, the participant was instructed to move on to the next task at their own pace, until they finish the last task on the list, or want to stop trying. It was made clear that during the task section, the experimenter would not interfere or offer help. The exception was when the participant feels sick: they could sign the experimenter and the experiment would be aborted.

Next, the experimenter showed the questionnaires and went through all items to ensure that each question was understood. It was told that the whole experiment aimed to investigate user experience. The participant was then given a pen and clip board, so that they could take notes during the task section if needed.

Before the task section, the participant was informed which of the assistant they would interact with (either 'Echo' or 'Alexa', half of the participant start with 'Echo'). Then, the experimenter acted as if to mute the 'not involved' smart speaker, while covertly muting both, in order to utilize Wizard of Oz techniques.

Once the task section started, the participant would interact with the 'assistant' from the sitting position shown in Figs 1 and 2. To each valid question asked, the experimenter plays a corresponding audio file, thus faking autonomous response by the assistant. For each question, three to five various response audio files were prepared. For instance, each time the participant retries the task 'Roll a dice', the experimenter would play another file which gave a different answer than the previous one. Should the participant stumble or fail to make valid question, the experimenter would play one of the response files that ask for repeating, e.g., 'Could you say that again?' For tasks involving smart light bulb control, a Philips Hue

was controlled by the experimenter via smartphone.

The task section ended when either the last task was finished, or if the participant signed that enough attempts were made. The experimenter then invited the participant to a table away from the experiment setup to answer the questionnaire, meanwhile acted as if to mute the smart speaker. Upon finishing the questionnaires, the participant was given the second task list, and the experiment again faked as if to activate the assistant involved. From there, the second task section was carried out same as the first one. Upon the second set of questionnaires being answered, the experiment entered the free choice period.> To start the free choice period, the experimenter used the excuse of needing to print extra documents in order to justify leaving the room. Before leaving, the experimenter covertly reactivated the microphones of the smart speakers, which meant that the participants could interact with the real Echo Dot and Echo Plus during the free choice period. Interactions during the free choice period were recorded by the experimenter’s computer microphone.
