# Supplementary material for: Intrinsic motivation in virtual assistant interaction for fostering spontaneous interactions
Source: PLoS One. 2021 Apr 23;16(4):e0250326. doi: 10.1371/journal.pone.0250326 (PMC8064575; doi:10.1371/journal.pone.0250326)
Supplement: Attachment — Submitted filename: Response to Reviewers.docx [file pone.0250326.s010.docx]

Oct. 14^th^, 2020

Dr. Stefano Triberti

Academic Editor

*PLOS ONE*

Dear Editor and Reviewers:

Thank you for reviewing our work and offering detailed comments that exposed the weakness and flaws in the submitted manuscript. We agree with both reviewers that the manuscript was too abstract and lacked thorough clarification. We sincerely appreciate all the comments and are confident to improve our work.

With the current revised version, we addressed each comment received. After careful considerations, we decided to rewrite Chapter 2 Introduction and Chapter 3 Method, rather than patching everything up. We also added a paragraph in before Section 2.1 to briefly introduce the structure of our manuscript. The current version presents less abstract theoretical construct, clearer explanations of experimental settings and procedures, additional citations to support our arguments (e.g. on classification of activity and motivation), and a polished General Discussion. We believe the current version is more coherent and informative.

Regarding data disclosure. We apologize for misunderstanding the PLOS requirements. We now disclose all data in the form of Supporting Information.

Again, thank you very much for reviewing and commenting our work. We are looking forward to your feedback and further discussion.

In the ensuing paragraphs, we will cite and respond to the reviewers’ comments.

Reviewer 1

Comment 1

*The authors present the design and results of two experiments; the findings from the first experiment were used to inform the design of the second one, to my understanding. If that is the case, this should be clearly mentioned in the abstract and the introduction sections. Each Experimental section should be then renamed (instead of Experiment 1/2, place a more informative title). Moreover, the group design of these two experiments should be justified. Why each group design was used in each experiment. Is it a 2x2 design, since you investigate the effects of two variables? A justification would also help for comparison and reproducibility.*

Exactly as you pointed out, findings from the first experiment were used to inform the design of the second one. We renamed the second experiment ‘Verification experiment on uncertainty’s effect’(P. 23, ll. 482).

We clarified and justified group design of the two experiments in Section 3.2 (P. 14-15, ll. 291-299) and Section 6.1 (P. 24, ll. 495-496 & ll. 504-506), respectively.

Comment 2

*The hypothesis is clear and defined based on the theory discussed in Section 2. However, more references are needed to justify e.g., (Line 98) Since the explicit goal of exploration is learning, it can be concluded that uncertainty is responsible for non-intrinsically motivated activities. Moreover, a clear but short description of the tasks used should be also included in the text and not only in the tables. Why such tasks were selected? Maybe parts of Section 3.2.5 could be moved to the introduction as background knowledge.*

We rewrote the theoretical construct and hypothesis part. In the revised version, the role of uncertainty is introduced borrowing the Bayesian model of sensory perception [1] in Section 2.2 (P. 8, ll. 154-160 & P.9, ll. 171-175). The hypothesis on uncertainty is discussed in Section 2.4 (P. 10-11).

A brief introduction to tasks and why they were selected is now presented in Section 3.2, Manipulation of variables.

Comment 3

*Were the participants asked to carry out the tasks in the given order, as presented in Tables S1 and S2? As mentioned in the first comment, the design of the groups must be explicitly defined and clearly described. How were the participants assigned to groups?*

The participants were asked to follow the order as shown in Tables S1 and S2. Participants were randomly assigned to either large or small uncertainty group.

To better explain experiment design and implementation, we rewrote Chapter 3, Method and added a S4 Appendix, which serves as a step-by-step recipe for duplicating our experiment.

Comment 4

*Table 1 is not informative. Speech recognition is not mentioned before that point and its not clear to me what Cases 1-4 are and how they are used in the experiment.*

We removed Table 1 and explained the cases in Section 6.1 (P. 23-24, ll. 487-494). The essential point is that, behind the assistant’s ‘Sorry’ response exist two possible cases: whether the user’s utterances are correctly or incorrectly recognized. We argued that these two should and can be differentiated. Recent studies also stressed the importance of clarifying the assistant’s limitations [2, 3, 4], as we will present in the updated General Discussion (P30, ll. 630-632).

The idea of prompting informative responses originated from our observation that Alexa would prompt the same type of ‘Sorry’ response regardless of whether she recognized the user’s utterances correctly. We were able to learn about this difference thanks to her browser-based console. From there, we proposed that uncertainty can be reduced by giving informative responses.

Comment 5

*I am not sure about the categorization of the user utterances into intrinsic and non-intrinsic. for example, "Translate something" could be considered intrinsic or not based on the way it is expressed, or not? e.g., (Can you) translate this for me? Is it test or pastime activity?*

Classification criteria of user utterances are now presented in Chapter 4 Results (P. 18-20, ll. 380-415).

‘Translate something’ is not extrinsic when the user possess already knows the meaning of that ‘something’, because asking so does not raise productivity. This is the case in our observation because the participant who requested English-to-Japanese translation spoke fluent Japanese. We argue that such requests were to reduce boredom, thus intrinsically motivated. Conversely, if the user did not know the meaning before asking, it should count as extrinsic.

On the other hand, asking ‘Can you translate the sentence for me?’ or ‘What languages do you speak?’ points to the assistant’s capability, which counts as exploration activity, hence epistemic.

Comment 6

*Experiment 2 has to do with explainability. Maybe a few references of Explainable AI and how it affects user motivation should be included.*

Thank you very much for the hint. We found and added citations which mentioned the benefit and need to make future AI explainable. Please see Section 9.1 (P. 30, ll. 622-632).

Comment 7

*The manuscript needs proofreading and many English language edits.*

We plan to have a professional proofreading agency to check our manuscript before the final resubmission.

Reviewer 2

Comment 1

*This is a complex study, and I had some difficulty following all the threads of reasoning until I reached the second, more simplified, experiment. The theoretical construct as laid out in section 2 involves the interrelations of multiple concepts, and it is described mostly in abstract axioms. It would help to tie the abstractions to possible real-world scenarios, both to clarify and to motivate the construct.*

We agree that the theoretical construct was highly abstract and lacked coherence. In the revised version, we added real-world examples as well as illustrations to better explain the abstract axioms.

P. 6, ll. 106-113 now uses the example of ‘booking a flight ticket’ to demonstrate how users weigh capability expectation against expected task difficulty.

Section 2.2, P. 7-9 now uses illustration to visualize the interplay between expectation, observation, perception and uncertainty.

Section 2.3, P. 9, ll. 181-187 now real-world examples to define high versus low expectation.

Section 2.4 now uses illustrations to explain the hypothesis (situational effect by uncertainty). Small versus large uncertainty is also explained using task examples in Section 3.2, P.13, ll. 269-273.

Comment 2

*Some terms and concepts are invoked without definition. These include ‘robot,’ ‘complete interaction,’ ‘meaningful interactions,’ and ‘fitting slots.’ Also, the terms ‘subjective’ and ‘objective’ for the data collected in the experiments seem somewhat judgmental. Less value-laden and more descriptive terms would be ‘self-reported’ and ‘observed behavior’.*

Thank you for the comments. We made definition to these concepts and adopted the terms ‘self-reported’ and ‘observed behavior’ in the revised manuscript.

Robot: P. 3, ll. 52.

‘Complete, meaningful’ interaction: P. 18, ll. 369-370.

Filling slot: P. 31, ll. 641-647

Comment 3

*The Methods seem basically fine, although minor clarifications are needed. The role of the two devices (Echo and Dot) in the experimental setups is not stated as clearly as it could have been, especially for Experiment 1. Also, more information is needed about participant demographics and the times when the experiments were performed.*

P. 12-13, ll. 250-257 gives a concise introduction on the roles of devices. Experimental settings are now reported in detail, in Section 3.4 and S4 Appendix.

We added information on participant demographics and experiment times: P. 16, ll. 333 and P. 25, ll. 512 for the two experiments, respectively.

Comment 4

*The Discussion is theoretically informed and clearer than the introductory sections. The comments about the gratifications of uncertainty resolution (“second rewards”) are quite insightful.*

We rewrote Chapter 2 Introduction for clarity.

In the revised Chapter 9 General Discussion, we elaborated on the mechanism of second rewards (P. 30, ll. 624-632).

Comment 5

*The written English is virtually grammatically flawless. However, in the first two major sections of the paper the writing is highly abstract and for that reason, sometimes difficult to follow.*

We plan to have a professional proofreading agency to check our manuscript before the final resubmission.

The revised Chapter 2 Introduction now includes real-world examples and illustrations to reduce abstractness.

Section 2.2, P. 7-9 now uses illustration to visualize the interplay between expectation, observation, perception and uncertainty.

Section 2.3, P. 9, ll. 181-187 now real-world examples to define high versus low expectation.

Section 2.4 now uses illustrations to explain the hypothesis (situational effect by uncertainty). Small versus large uncertainty is also explained using task examples in Section 3.2, P.13, ll. 269-273.

Comment 6

*P 2, ll 19-20 “With the growing utility of today’s conversational virtual assistants, the importance of user motivation in human-robot interaction is becoming more obvious.” (I’m used to distinguishing virtual assistants from robots. What is your definition of a robot?)*

We should have been more careful about using the term ‘robot’. Actually, we only discussed about those robots that interact with users as conversational agents. Industrial robots such as one used for manufacturing (welding, painting, etc.) are not of our interest.

In the revised Chapter 2 Introduction, we clarified the scope of robot included in our discussion (P. 3, ll. 52.). In addition to the citations in HRI, we added recent studies on human-AI interaction to stress the importance of intrinsic motivation in designing virtual assistants.

Comment 7

*L 35 (“smartness” = “likeability”? This is not always true for people. Why should we assume it for CVAs?)*

We agree with your opinion, smartness is not necessarily equivalent to likeability. After careful consideration, we realized that ‘general smartness’ is not our focus. Our focus is the non-usefulness related qualities of virtual assistants, which we believe has much to do with user’s intrinsic motivation. Several recent studies on ‘voice assistant’ usage and motivation focused on ‘affective experience’ (as opposed to ‘task-oriented experience’) and ‘hedonic benefits’ (as opposed to ‘utilitarian benefits’), advocating that researchers should ‘…go beyond the task-oriented approach of traditional HCI and focus on hedonic aspects such as fun and pleasure.’ [5, 6] We cited such recent attention and proceeded to argue the novelty of our current research in comparison, as presented in the revised Chapter 2 Introduction (P. 2-3).

Comment 8

*P 3, ll 46-48 “Intrinsic motivation should be the next focal issue in virtual assistant interaction (and indeed in HRI generally) because it is crucial for enhancing the likability and the perceived smartness of future smart products.” (The causality goes in the opposite direction, no?)*

Yes, the causality should go in the opposite direction. We have adjusted accordingly (P. 3, ll. 58).

Comment 9

*L 55 “circumstances the user (in general, the agent)” (Calling the user of an AI agent the ‘agent’ is confusing. What’s wrong with ‘user’?)*

Thank you for the comment. We adapted to use the term ‘user’ in general and used ‘participant’ in context of experiment. We initially used ‘agent’ because it sounded more ‘generalized’ (from P. 4, ll. 67).

Comment 10

*Ll 56-57 “Eventually, we wish to anticipate and even shape behavioral strategy, thus fostering spontaneous interactions. Such findings would be valuable both for the academic community and for industry.” (Spell out what the value would be.)*

As mentioned in earlier parts of Introduction, our research is valuable for covering unstudied mechanism of intrinsic motivation in the specific context of virtual assistant interactions. As for the industry, we believe our findings offers design guidelines for better product engagement, as discussed in Section 9.2 Application to virtual assistant design.

In the revised manuscript we opted to be concise, stating that one can enhance product engagement through fostering user interactions (P. 4, ll. 66).

Comment 11

*L 58 “We define a spontaneous interaction as any action that is taken by agents of their own will.” (In your experiments, ok, but not in general, since users ask Alexa for information for extrinsic motives “of their own will” all the time.)*

We agree that in general, in everyday life, interactions happen of the user’s free will. On the other hand, in cases of laboratory studies, we used the term ‘spontaneous’ to distinguish such interactions from the ‘interactive tasks’ assigned to the participants. This is now clarified in Chapter Introduction 2, P. 4, ll. 70-76. In Sections 2.1 and 2.3, we mentioned another layer of the nuances: The opposite of ‘initiating a spontaneous interaction’ can be seen as when the user has an intention but ‘didn’t bother’ to use the assistant (P. 6, ll. 106-113).

For now, we are keeping ‘spontaneous’ in the title. We would like to have further discussion with you and see if adjustment is needed.

Comment 12

*P 4, ll 76-78 “Normally, after the learning process, agents can re-evaluate the assistant’s capability and their own action strategies by asking whether their commands met with compliance. In the presence of too much uncertainty, such re-evaluation becomes difficult.” (Why is that, exactly? The abstractness of some of the statements in this section makes them difficult to process, e.g., “An interaction suffers from information loss at the interface” and “uncertainty diminishes the effect of expectation.” Perhaps these are truisms in the authors’ discipline, but as a potentially interested reader from outside the discipline I find them puzzling. More explanations and hypothetical examples would help.)*

Thank you very much for reporting the obscurity. In the first submission we borrowed puzzling terms and concepts from multiple theories without sufficient definition.

In the revised manuscript we added examples and illustrations, please see response to Comment 1.

As for issues brought up in this comment, ‘effects of uncertainty’ is now addressed in Section 2.2 (P. 8, ll. 154-160) and ‘information loss at the interface’ is addressed in Section 2.1 (P. 5, ll. 102).

Comment 13

*P 5 ll 88-89 “research has shown that an intelligent robot is perceived as more likable [10].” (Independent of anthropomorphism, size, mobility, etc.?)*

After examining the literature, we found out that perceived intelligence is not independent of other factors to decide likeability. We decided, as mentioned in the response to Comment 7, not to directly link ‘intelligence’ to ‘likeability’. In the revised Section 2.1, we now focus on how expectation of capability can affect user’s motivation. We argue that if the expectation of capability is too low, the user will believe it is futile to interact, thus will not be motivated to interact (P. 6, ll. 106-118).

Comment 14

*Ll 91-92 (Your “clarification” here is confusing. Reword or omit.)*

It is now omitted (the equivalent context is found at P. 6, ll. 113).

Comment 15

*Ll 97-99 “Generally, such disruption will lead the agent to re-explore the capability of the virtual assistant. Since the explicit goal of exploration is learning, it can be concluded that uncertainty is responsible for non-intrinsically motivated activities.” (How does this follow logically?)*

The role of uncertainty is introduced borrowing the Bayesian model of sensory perception [1] in Section 2.2 (P. 8, ll. 154-160 & P.9, ll. 171-175). The hypothesis on uncertainty is discussed in Section 2.4 (P. 10-11).

Comment 16

*P 6, Ll 102-107 “Cue-integrating theory states that the larger the uncertainty, the less reliable the evidence [12]. Therefore, the resultant expectation is always closer to the more reliable evidence. When observations suggest that the virtual assistant is of low capability, small uncertainty will draw expectation towards observation, because the observation will be considered accurate. However, large uncertainty reduces the impact of the observations … As a result, we believe that when expectation is low, large uncertainty leads to higher expectation, and thus benefits motivation more than small uncertainty.”*

*(I’ve read these sentences over several times and still don’t understand them. At least five different concepts are invoked in this subsection (expectation, degree of expectation, uncertainty, degree of uncertainty, motivation [intrinsic and extrinsic]). The relationships among them should be laid out more systematically. “Inconsistent performance by the virtual assistant gives rise to uncertainty;” ok. “Capability expectation is based not only on the agent’s observations, but also on the agent’s prior belief in the assistant’s capability;” ok, although this raises the question: What gives rise to the prior beliefs, if not the agent’s observations? What determines high vs. low expectations in the real world? Also, what determines “small” versus “large” uncertainty?)*

Although it is difficult to reduce number of involved concepts, the revised Introduction on these concepts are now supported by examples and illustrations. We also limited the number of theories, for instance, we omitted cue-integrating theory can be substituted by detailed breakdown on the Bayesian model (P. 7-8, ll. 133-153).

Regarding the raised questions, *current* observation gives rise to ‘prior beliefs’ for *future* interactions. Definition of high vs. low expectation and small vs. large uncertainty is now presented in Sections 2.3 and 2.4 using real-world examples (P. 9, ll. 181 & P. 10, ll. 195).

Comment 17

*Ll 113-116 “we hypothesize that intrinsic motivation is affected by the interacting variables expectation and uncertainty as follows: High expectation has positive effects on intrinsic motivation. Uncertainty has beneficial effects when expectation is low and harmful effects when expectation is high.” (What possible real-world scenarios do these hypotheses map onto?)*

The hypotheses are now introduced in Sections 2.3 and 2.4.

Real-world scenario:

Assistant A is stable. It excels at easy tasks such as setting timers, but is observed to always fail in difficult tasks, such as answering trivia questions.

Assistant B is unstable. It sometimes fails in setting timer correctly, but can cope with more difficult tasks, say, 40% of the time e.g. when asked trivia questions.

In this situation, both assistants appear to have low capability. We hypothesized that it is more rationale to predict that Assistant B (large uncertainty) can accomplish difficult tasks, therefore has better potential to satisfy the user’s intention than Assistant A (small uncertainty)

An equivalent but concise argument is made in Section 2.4, P. 10, ll. 207-211.

Comment 18

*P 7, ll 141-142 “S1 and S2 Tables” --> “Tables S1 and S2”*

Thank you for pointing out the problems. Addressed (from P. 13, ll. 263).

Comment 19

*P 8, l 155 “The participant sits 50 centimeters away from the table.” (Give distance in inches also.)*

Added.

Comment 20

*Pp 8-9 (Consider reversing the order of presentation of sections 3.2.1 and 3.2.2. Based on lines 158-160, I expected uncertainty to be discussed first. Or else reorder presentation of terms in lines 158-160 to mention expectation first.)*

In the revised Section 3.2, Manipulation of variables, uncertainty is now discussed before expectation.

Comment 21

*P 10, line 188 “Thirteen healthy students from the University of Tokyo participated in the experiment” (How is their health relevant? But give age range and gender breakdown. Also, indicate when the experiments were conducted. I’m surprised that none of the students was familiar with virtual assistants. In the US, the Amazon Echo has been around since 2015.)*

Health is now omitted since it is not relevant. Age range, gender and experiment time are added, P. 16, ll. 333 and P. 25, ll. 512.

We screened participants during recruiting, tech savvy students were excluded.

Comment 22

*L 192 (How much is 1000 Japanese Yen in US$?)*

Added (P. 16, ll. 338).

Comment 23

*L 203 “can be found in S4 Spreadsheet” --> “can be found in Spreadsheet S4”*

Addressed (from P. 17, ll. 345).

Comment 24

*P 11, ll 226-227 We counted the numbers of complete interactions observed during free-choice period” (How did you operationalize a ‘complete interaction’?)*

The definition is now clarified (P. 18, ll. 369-370), borrowing the hints from Comment 30.

Comment 25

*P 12, l 235 “The participants could choose freely to interact with either assistant” (They had different wake words, then, one assumes. What were the wake words? Also, how were the two assistants introduced to the subjects?)*

Roles of assistants including wake words are now introduced in Sections 3.1 and 3.2 (P. 11, ll. 229 & P. 14, ll. 285). To briefly answer the question: the wake words were ‘Alexa’ for the high-expectation assistant and ‘Echo’ for the low-expectation assistant.

The experiment instructions and other procedures are introduced in Appendix S4.

Comment 26

*L 242 “These two categories of activities were driven by intrinsic and non-intrinsic motivation, respectively” (Explain your criteria for making these classifications. It’s not enough just to cite sources.)*

Classification criteria are now presented in Chapter 4 Results (P. 18-20, ll. 380-415).

Comment 27

*P 14, ll 271-272 (A note on terminology. Instead of ‘subjective’ and ‘objective’, less value-laden and more descriptive terms would be ‘self-reported’ and ‘observed behavior’.)*

Thank you for the advice. We adapted to use the descriptive terms (from P. 15, ll. 301).

Comment 28

*P 16, l 329 (Give age range and gender breakdown of participants. Also, when did Experiment 2 take place in relation to Experiment 1?)*

Information added in Section 6.2. The second experiment took place 7 months from the first one.

Comment 29

*Ll 339-340 “Only two participants made conversation with the large-uncertainty virtual assistant; the other participants only interacted with the small-uncertainty virtual assistant.” (So this is how the two devices were differentiated – was this also the case in Experiment 1? Their roles should be clarified.)*

Roles of the assistants are now introduced in Sections 3.1 and 3.2.

Please see P. 12-13, ll. 250-257 for a brief introduction on their roles.

Comment 30

*L 344 “The number of meaningful interactions” (In what sense are they “meaningful”? A better characterization might be “complete exchanges, consisting of an initiation by the agent and a response by the assistant.”)*

Thank you for the advice. We redefined ‘complete interaction’ borrowing this comment (P. 18, ll. 369-370).

Comment 31

*P 20, ll 404-407 “Non-intrinsic motivation is derived from the need to achieve an external goal, which in the context of virtual assistant interaction involves testing and making trials. Intrinsic motivation is derived from the expected enjoyment of the action itself; it is responsible for activities such as play”*

*(Where does seeking answers to self-generated information questions, such as “what is tomorrow’s weather?” fall according to this classification? They are not asked to test or make trials, nor to play, but rather to gain information for one’s own use.)*

Thank you for pointing out the missing part. Here we only discussed epistemic (test and trial) and left out extrinsic motivation. In general, asking for weather should be considered extrinsic, hence non-intrinsically motivated. However, asking for the weather in Tokyo is an exception to this.

During the experiment instructions, we have demonstrated how to ask for weather information using the assistants. As response, the assistants reported the weather in Tokyo (Currently it is…, tonight…, tomorrow…) Therefore, unless the participant forgets about the demonstration, there is no rationale to test the assistants’ capability of weather forecasting, hence epistemic motivation should be ruled out. Furthermore, since the weather was already known, asking again does not provide new information (no pragmatic/utilitarian value), thus ruling out extrinsic motivation.

If the participant asked for the weather elsewhere (we observed several weather inquiries about Munich and Vienna). This provides utilitarian value, therefore counts as extrinsically motivated.

In general, if the user is exploring whether the assistant is capable of checking weather, the action should be considered epistemically interacted (counts as learning, which enables utilitarian usages in the future). If the user is certain about the capability and asking would provide useful information for the user, then it should be seen as extrinsically motivated. The action promises utilitarian value and is driven by a separable goal. Borrowing Friston’s terminologies, the former case falls into exploration, the latter falls into exploitation.

Classification criteria are now given in Chapter 4 Results (P. 18-20, ll. 380-415).

Comment 32

*P 21, ll 426-430 “The pleasure perceived when the work finally makes sense is termed the “second reward” [20], as opposed to the first reward from simply receiving the information. The informative response strategy works the same way. It provides the agent with crucial information to resolve the uncertainty induced by the first few words when the assistant reports that it fails to cope.”*

*(I very much like the analogy to uncertainty resolution in artistic works, but the last sentence as worded does not convince me that the two situations are very similar. I think you can make a stronger case by rewording it.)*

We reworked the scenario of hearing an informative response, pointing out that it facilitates the process of making sense and also provide users with guidance for future interactions (P. 30, ll. 624-632).

Comment 33

*P 22, l 436 “Nevertheless, if we wish to apply such knowledge to foster intrinsically motivated interaction, it is effective to create a second reward.” (Elaborate: create a second reward by doing … ?)*

We made adjustments and emphasized the effectiveness of informing the limitations of the assistant after the inevitable ‘Sorry’ (P. 30, ll. 637-639).

Comment 34

*L 439 “recognize users’ intentions by fitting slots.” (Explain or paraphrase this term.)*

The term should be ‘filling slot’. It is now explained in P. 31, ll. 641-647. Here we used the example of Amazon Echo. Slots are variables that are embedded in applications to serve the user’s requests. They are ‘filled’ by picking up information from user utterances. For instance, a flight booking application typically expects to fill slots such as ‘destination’, ‘date’, etc.

References

1. Yanagisawa H. A computational model of perceptual expectation effect based on neural coding principles. J Sens Stud. 2016; 31(5): 430-439.
2. Weber P, Ludwig T. (Non-) Interacting with conversational agents: perceptions and motivations of using chatbots and voice assistants. Proceedings of the Conference on Mensch und Computer. 2020; 321-331.
3. Amershi S, Weld D, Vorvoreanu M, Fourney A, Nushi B, Collisson P, Suh J, Iqbal S, Bennett PN, Inkpen K, Teevan J, Kikin-Gil R, and Horvitz E. 2019. Guidelines for Human-AI Interaction. In Proceedings of the 2019 CHI Conference on Human Factors in Computing Systems. 2019; 1-13.
4. Brandtzaeg PB, Følstad A. Why people use chatbots. International Conference on Internet Science. 2017; 377-392.
5. McLean G, Osei-Frimpong K. Hey Alexa… examine the variables influencing the use of artificial intelligent in-home voice assistants; Comput Human Behav. 2019; 99: 28-37.
6. Yang X, Aurisicchio M, Baxter W. Understanding affective experiences with conversational agents. Proceedings of the 2019 CHI Conference on Human Factors in Computing Systems. 2019; 1-12.
