# Supplementary material for: Intrinsic motivation in virtual assistant interaction for fostering spontaneous interactions
Source: PLoS One. 2021 Apr 23;16(4):e0250326. doi: 10.1371/journal.pone.0250326 (PMC8064575; doi:10.1371/journal.pone.0250326)
Supplement: Attachment — Submitted filename: Response-to-Reviewers_PONE-D-20-16053.docx [file pone.0250326.s011.docx]

Mar. 28^th^, 2021

Dr. Stefano Triberti

Academic Editor

*PLOS ONE*

Dear Editor and Reviewers:

Thank you for feedback and comments on our revision. We addressed each comment and also had a proofreading team check the manuscript. In the ensuing paragraphs we cite and respond to the reviewers’ comments. Please refer to the line numbers to locate modifications/revisions. Also, please note that we have replaced “chapter” with “section”, since “chapter” is rather used in books.

**Reviewer 1**

Comment 1

*After the first sentence of the abstract, a proper definition of user motivation should be introduced, in the context of virtual assistants, i.e., is it related to the amount of time users interact with such assistants? or is it when users use it just for fun as you state in Line 66?*

Lines 22-25. We added brief definition of intrinsic and non-intrinsic motivation.

Comment 2

*Paper outline - starting Line 81: make sure you use the appropriate tense, i.e., "we first introduce" instead of "we will first introduce", "we summarize our findings ... and propose suggestions" instead of "we will summarize our findings ... and proposed suggestions"*

Corrected. Starting from line 80.

Comment 3

*A reference is needed for the statement at the paragraph from Line 93.*

Line 91. The reference here is:

Krippendorff K. The semantic turn: A new foundation for design. 1st ed. Boca Raton, FL: CRC Press; 2005.

Comment 4

*Line 108: ("I need to book a flight")*

Line 106. We corrected this sentence.

Comment 5

*After Line 118, an example of "interact for fun" with a virtual agent would be helpful at this point. It can be one of the tasks used in the study to help readers understand.*

Line 117. Tasks used in the study were primarily non-intrinsically motivating. We added an example: chatting.

Comment 6

*In your analysis, several tests are used (t-tests, anova, etc.). It would be useful to add one sentence for why each test was used.*

Lines 346 & 432. We added explanation for our choices. Two-way ANOVA was chosen to test interaction effect (expectation and uncertainty). T-test was chosen to investigate effects on single variables.

Comment 7

*In general, avoid using subjective language for the analysis of the results. To be specific, I would not use the phrase "unexpected findings" for the first experiment. On the contrary you could state it in a more objective way, stating that since the hypothesis was rejected, the model was revised and a verification experiment was designed.*

Line 442. We corrected subjective wording.

**Reviewer 2**

Comment 1

*Ll 32-34 “The results also suggested suppressive effects by uncertainty on intrinsic motivation, though we had not hypothesized before experiments.” (Sentence is somewhat unclear, also ungrammatical.)*

Line 33. We removed this statement. Instead, we clarified that the first hypothesis was not supported.

Comment 2

*Ll 51-52 “and human-robot interaction (HRI; though only those robots who interact with users as conversational agents are of our interest),” (Why mention HRI in this paper at all? It creates potential confusion, it seems to me. If the implications of your experiments extend to HRI, you could just mention that towards the end, in addressing the broader implications of the study. Or omit it entirely.)*

Line 58. We omitted HRI.

Comment 3

*Ll 62-63 “Unfortunately, virtual assistants are typically treated as autonomous tools by adult users” (The evaluation ‘unfortunately’ seems inappropriate here. More neutral: “However,”)*

Lines 45 & 61. Thank you. We checked the document and corrected subjective languages.

Comment 4

*L 85 “a two-by-two experiment, which will be introduced in Chapter” (It is conventional to use the simple present tense, rather than the future tense, in describing the contents of the paper.)*

Thank you. We addressed the tense issue starting from line 84.

Comment 5

*L 97 “When the observation is unexpected” --> “When what is observed is unexpected,”*

Line 95. Corrected.

Comment 6

*Ll 134-135 “human’s posterior perception” (What is that? Perception “after the fact”? Define the term. The word ‘posterior’ has other meanings in English that could cause confusion. Less ambiguous terms would be Latin ‘a posteriori’ vs. ‘a priori.’ Or you could replace posterior with “after the fact.”)*

Starting from line 135. We replaced these terms with ‘a posteriori’ and ‘a priori’.

Comment 7

*L 138 Please briefly explain what the “Bayesian Estimator” is and what it does.*

Line 140: We now briefly explain each term in the *Bayesian estimator* (Equation 1) in general sensory perception scenario, before moving to the context of assistant interaction.

Comment 8

*Ll 157-160 “Although the original model distinguished between uncertainty and noise, in that noise flattens the likelihood distribution whereas uncertainty flattens the prior distribution, we consider ‘noise’ as the uncertainty perceived by the user over the course of multiple interactions.” (What is the reasoning behind this change?)*

Line 162. We removed this inappropriate statement about ‘noise’, since we did not introduce ‘noise’ to our experiment. According to the original model by Yanagisawa et.al. [13], ‘noise’ is defined as the impediment to the ‘information gathering’. ‘Noise’ would be introduced, if we deliberately made one of the agents ‘speak’ through electric buzz/hum-which we did not.

Throughout the task sections, inconsistent performance induces uncertainty. Such uncertainty eventually flattens the *a priori* distribution, right before the free-choice period. In our experiment, uncertainty is considered the only cause of flattened *a priori* distribution.

Comment 9

*L 196 “if not non-existing” --> “if not non-existent”*

Line 198. Thank you. Corrected.

Comment 10

*Ll 207- onwards. I was following the exposition well up to this point, but I’m still struggling to wrap my head around all the variables in the Discussion of figure 2. It’s not just expectation level and uncertainty level that interact here, but also task difficulty – 3 variables, each with two values. It would be helpful to make a hypothesis about uncertainty independent of the other two variables first, like you do with expectation, before adding the other variables into the mix. … After reading a little further, I also wonder: Since you assume that expectation is affected by uncertainty/inconsistency, can uncertainty be separated from expectation with conversation agents? If not, are you really analyzing two separate constructs? On a conceptual level, I question if they are logically independent variables.)*

Line 164. Two points should be addressed in this comment.

1. Regarding the third variable, task difficulty.
2. Regarding whether uncertainty and expectation are logically independent variables.

In response to 1: Task difficulty is not considered as a variable; it is a benchmark, which the expectation of capability is compared with. As explained in ll. 211, task difficulty is equal to least ‘capability’ required to cope with a task.

In response to 2: We need to clarify the definition of expectation. Using Fig 2, expectation is defined as the mean of probability distribution, whereas uncertainty is the variation. This definition is borrowed from the previous work of Yanagisawa et.al [13]. Therefore, expectation is not affected by uncertainty; these two are independent variables.

To dispel the confusion, consider the following structure:

a) The user intends to use the assistant to book a flight.
b) Before talking to the assistant, the user estimates whether the assistant is capable of booking a flight. (Comparing expectation of capability against the benchmark, i.e., task difficulty.)
c) The estimated outcome can be anywhere between ‘The assistant can do this’ or ‘I don’t think it can…’

The estimated outcome is indeed affected by both expectation and uncertainty. Important is, the estimated outcome and expectation of capability is two different things.

Comment 11

*Ll 220-221 “To sum up, we hypothesized a positive effect by expectation on motivation, and an interaction effect by expectation and uncertainty on motivation” (Is there any direct hypothesized effect of uncertainty on motivation? It seems that you get to this in the revised experimental protocol. Why not hypothesize it up front?)*

Line 222. In our research, we did not realize that uncertainty might have effects on motivation by itself until we analyzed data from the first experiment. The finding from the first experiment was essential for us to propose the revised model. Therefore, we insisted to present both experiments in the natural order.

Comment 12

*L 237 “the participant could interact with the real Amazon Echo assistants” (In what language?)*

Line 240. In English.

Comment 13

*L 253 “‘sculptured’” (The word you’re aiming for is “sculpted,” I think, but “constructed” might be better.)*

Line 255. Thank you. We now use ‘constructed’.

Comment 14

*L 314 “a Philips Hue” (needs another word. Bulb? Bridge?)*

Line 315. A Philips Hue LED bulb.

Comment 15

*Ll 394-396 “However, Weber [et.al --> et al.] [7] classified interactions which serve the purpose to ‘test the conversational agents’ understanding of language and to push it to its limits’ as ‘trolling’, which neither falls into entertainment nor utilitarian uses.” (Actually, malicious entertainment is one of the main reasons for internet trolling, according to studies that have interviewed trolls. But this usage with conversational agents seems more benign. Be aware that the word trolling normally has quite negative connotations, though. The next sentence about Friston et al. is more the perspective I think you want to adopt.)*

Line 395. We removed the contents about trolling, since the ensuing taxonomy by Friston suffices. Although Weber et.al. used the word ‘trolling’ in the context of conversational agent interactions, we do realize that it is a negative expression

Comment 16

*L 452 “when a precise goal is extant” --> “when a precise goal exists”*

Line 453. Corrected.

Comment 17

*L 456 “When uncertainty is [extant --> exists]”*

Line 457. Corrected

Comment 18

*L 461-462 “when there is no precise goal, the author argued [that] epistemic value can dominate action selection.” (The author here is Friston et al.?)*

Line 462. Yes, the author is Friston et al [12].

Comment 19

*L 465 (Indicate source of the ‘original model’ in the label for Fig. 16.)*

Line 466. Added: reference Friston et al [12].

Comment 20

*L 564 “Participants on average interacted 5.6667 times” (Reduce number of decimal places in reporting results. Your sample size does not justify this degree of precision. Two decimal places are sufficient.)*

Line 545. Corrected.

Comment 21

*In Table S6a, the content of the right cell of row 8 is incorrect.*

Corrected.

Comment 22

*Table S6B, “The virtual assistant was made to appear unable to cope with tasks 3, 7, 9 and 12, and these responses were not” (word missing – “informative”?)*

Yes, we missed “informative”. Corrected.
